# Extensive horizontal gene transfer, duplication, and loss of chlorophyll synthesis genes in the algae

**DOI:** 10.1186/s12862-015-0286-4

**Published:** 2015-02-10

**Authors:** Heather M Hunsperger, Tejinder Randhawa, Rose Ann Cattolico

**Affiliations:** Department of Biology, University of Washington, Seattle, WA USA

**Keywords:** Chlorophyll synthesis, Horizontal gene transfer, Endosymbiotic gene transfer, Gene duplication, Algae, Protochlorophyllide

## Abstract

**Background:**

Two non-homologous, isofunctional enzymes catalyze the penultimate step of chlorophyll *a* synthesis in oxygenic photosynthetic organisms such as cyanobacteria, eukaryotic algae and land plants: the light-independent (LIPOR) and light-dependent (POR) protochlorophyllide oxidoreductases. Whereas the distribution of these enzymes in cyanobacteria and land plants is well understood, the presence, loss, duplication, and replacement of these genes have not been surveyed in the polyphyletic and remarkably diverse eukaryotic algal lineages.

**Results:**

A phylogenetic reconstruction of the history of the POR enzyme (encoded by the *por* gene in nuclei) in eukaryotic algae reveals replacement and supplementation of ancestral *por* genes in several taxa with horizontally transferred *por* genes from other eukaryotic algae. For example, stramenopiles and haptophytes share *por* gene duplicates of prasinophytic origin, although their plastid ancestry predicts a rhodophytic *por* signal. Phylogenetically, stramenopile *por*s appear ancestral to those found in haptophytes, suggesting transfer from stramenopiles to haptophytes by either horizontal or endosymbiotic gene transfer. In dinoflagellates whose plastids have been replaced by those of a haptophyte or diatom, the ancestral *por* genes seem to have been lost whereas those of the new symbiotic partner are present. Furthermore, many chlorarachniophytes and peridinin-containing dinoflagellates possess *por* gene duplicates.

In contrast to the retention, gain, and frequent duplication of algal *por* genes, the LIPOR gene complement (chloroplast-encoded *chlL*, *chlN*, and *chlB* genes) is often absent. LIPOR genes have been lost from haptophytes and potentially from the euglenid and chlorarachniophyte lineages. Within the chlorophytes, rhodophytes, cryptophytes, heterokonts, and chromerids, some taxa possess both POR and LIPOR genes while others lack LIPOR. The gradual process of LIPOR gene loss is evidenced in taxa possessing pseudogenes or partial LIPOR gene compliments. No horizontal transfer of LIPOR genes was detected.

**Conclusions:**

We document a pattern of *por* gene acquisition and expansion as well as loss of LIPOR genes from many algal taxa, paralleling the presence of multiple *por* genes and lack of LIPOR genes in the angiosperms. These studies present an opportunity to compare the regulation and function of *por* gene families that have been acquired and expanded in patterns unique to each of various algal taxa.

**Electronic supplementary material:**

The online version of this article (doi:10.1186/s12862-015-0286-4) contains supplementary material, which is available to authorized users.

## Background

**Figure 1:**
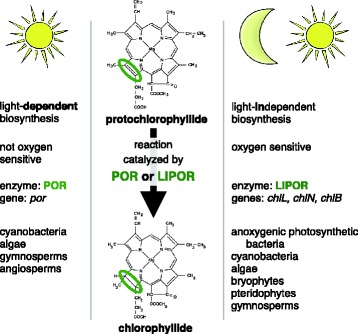
**Comparison of the POR and LIPOR enzymes.** The second to last step of chlorophyll synthesis can be catalyzed by either a light-dependent (POR) or light-independent (LIPOR) protochlorophyllide oxidoreductase (figure after [2,3]).

Chlorophyll *a* is synthesized entirely within the chloroplast, progressing in a series of enzymatic steps from the first committed precursor, 5-aminolevulinate, to the end product chlorophyll *a* [1]. The second to last step of this reaction sequence transforms the pigment protochlorophyllide to chlorophyllide via the reduction of a double bond. This step can be catalyzed by either of two non-homologous, isofunctional enzymes: the light-independent (LIPOR) or the light-dependent (POR) protochlorophyllide oxidoreductase (Figure 1) [2,3].

The evolutionary origins and occurrence of POR and LIPOR oxidoreductases differ. LIPOR first arose in anoxygenic photosynthetic bacteria, likely evolving from a nitrogenase [4,5]. Similar to nitrogenase in structure, this enzyme is comprised of one or two L-protein homodimers encoded by the *chlL* gene and an NB-protein heterotetramer encoded by the genes *chlN* and *chlB*. Also like nitrogenase, the LIPOR holoenzyme contains iron-sulfur clusters that confer sensitivity to oxygen [6-9]. In contrast, the POR enzyme arose in cyanobacteria [3], the first oxygenic photosynthesizers which are also thought to be responsible for the oxygenation of Earth’s atmosphere [10]. It is postulated that the POR enzyme arose under strong selective pressures for an enzyme that would be unaffected by oxygen [8,11]. The POR enzyme, encoded by the *por* gene, is a globular protein with high sequence similarity to other members of the short-chain dehydrogenase-reductase (SDR) family. Although the POR enzyme is insensitive to oxygen, it has its own Achilles’ heel. The enzyme is only active when its pigment substrate absorbs light [12] and thus, unlike LIPOR, POR cannot facilitate chlorophyll synthesis in the dark.

Endosymbiotic theory holds that chloroplasts originated when a non-photosynthetic protist engulfed and maintained cyanobacteria-like cells [13]. It is hypothesized that a single ‘primary’ endosymbiotic event generated the glaucophytic, rhodophytic and chlorophytic algae, as well as the viridiplantae ([14] but see [15,16]). In subsequent ‘secondary’ endosymbioses, the ancestors of euglenid and chlorarachniophyte algae each phagocytized and retained chlorophytes as plastids [17]. The origins of the cryptophyte, alveolate (e.g., dinoflagellate), stramenopile and haptophyte algae (collectively termed CASH) are less clear. Whereas nuclear genes show CASH host lineages to be polyphyletic [18], plastidial genes support a single, rhodophytic origin for their chloroplasts [19-22]. Synthesizing earlier views [23-25], the rhodoplex hypothesis describes any number of scenarios in which the initial CASH plastid was obtained via a secondary endosymbiotic event and transferred between or even within CASH lineages by tertiary and potentially higher order endosymbioses [26].

During the establishment of the proto-chloroplast, and in those organisms of serial endosymbiotic origin, most of the genes required for photosynthesis and organellar homeostasis were transferred from the endosymbiont to the host nucleus in a process known as endosymbiotic gene transfer (EGT). In fact, ~18% of *Arabidopsis* genes are of cyanobacterial origin [27]. In extant cyanobacteria, POR and LIPOR genes are each present as single copies. In eukaryotic algae, the gene encoding POR appears to have been transferred to the nucleus, whereas LIPOR genes remain chloroplast-localized when present (these three genes are lost in many photosynthetic organisms).

Regardless of coding location, genetic restructuring can also be catalyzed by horizontal gene transfer (HGT), the process whereby xenologs (foreign genes) are incorporated into the genome of an organism. Although HGT was once thought to occur rarely, it is now recognized as a potentially pervasive force in genetic restructuring [28]. Transferred genes can originate from a variety of sources, including phagocytized prey, symbioses, viral transfection, and potentially other sources not yet identified [29,30]. Recent, intense sequencing efforts across a broad representation of prokaryotes and eukaryotes have resulted in extensive documentation of horizontal gene transfer (e.g., [31-35]). For example, Archibald et al. [36] analyzed nuclear-encoded, plastid-targeted genes of a chlorarachniophyte and found that up to 21% of the studied genes were derived from foreign sources. Such high rates of HGT observed in microbes are hypothesized to result from a ‘gene transfer ratchet’, wherein a small probability of gene incorporation multiplied by many gene uptake events over time results in many orthologous as well as novel genes in microbial genomes [29]. Apart from chance incorporation, the successful integration of a transferred gene has been shown to be highest for genes: (a) involved in few or no protein-protein interactions [37,38]; (b) not involved in information processing (e.g., DNA replication, RNA transcription, and protein translation; [39]); and (c) expressed at low levels [40]. *Por* genes (see below) appear to fulfill these criteria.

Whether of ancestral or foreign origin, the duplication of resident genes serves as an additional source of genetic novelty. Gene duplication (i.e., the generation of gene paralogs) can potentially impact metabolic processes on several levels. Most simply, gene dosage is increased for the duplicated gene. Alternatively, mutations in regulatory regions or coding sequences can effectively partition a gene’s ancestral roles among the paralogs. If one copy mutates extensively, a novel protein may be generated. In many cases, genetic change arising from gene duplication provide an adaptive advantage and become fixed in the population (reviewed in [41]). Recent studies [42,43] suggest that the diatom *Phaeodactylum tricornutum* uses more than one POR enzyme for chlorophyll synthesis. Multiple *por* genes have also been annotated in three additional diatom genomes (*Thalassiosira pseudonana*, *Fragilariopsis cylindrus*, and *Pseudo-nitzschia multiseries* [44]). These observations generate many questions concerning POR duplication in diatoms as well as other algal species: Did extra *por* genes arise from HGT or gene duplication? Could the maintenance of redundant *por* genes account for the apparent loss of the genes encoding LIPOR (*chlL, chlN, and chlB*) in some chloroplast genomes [4,45,46]? Under what circumstances would the presence of both non-homologous, physiologically distinct protochlorophyllide oxidoreductases be advantageous to an organism?

In this paper we explore the nature of genetic novelty and genetic redundancy with respect to both the light-dependent (POR) and light-independent (LIPOR) enzymes that catalyze the penultimate step of chlorophyll synthesis. We find a reticulate *por* gene history within the algae, evidencing multiple horizontal gene transfer events including one that offers evidence of a close association of the plastids of haptophyte and stramenopile algae. Furthermore, we identify several *por* gene duplications and the presence of both ancestral and xenologous *por*s in some algal taxa. We also show a propensity for algae maintaining multiple *por* genes to lose their chloroplastic LIPOR genes (*chlL, chlN,* and *chlB*). Genetic redundancy, whether arising from non-homologous isofunctional enzymes, gene duplication, or horizontal gene transfer, fosters metabolic innovation. These data present an exciting opportunity to compare the fate of uniquely redundant protochlorophyllide oxidoreductase genes across evolutionarily close and distant lineages.

## Results and discussion

### POR protein phylogeny inference

**Figure 2:**
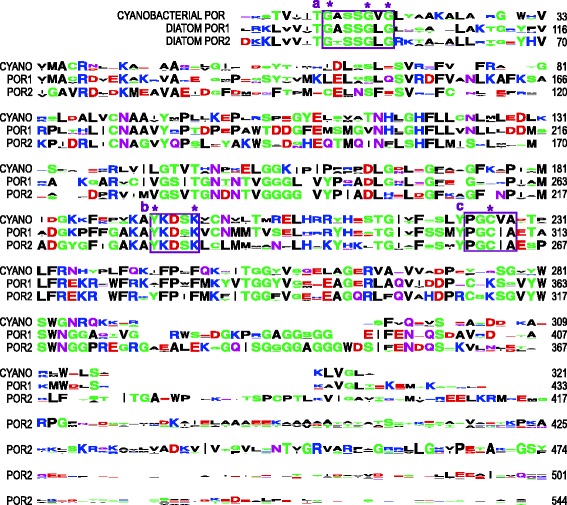
**Sequence logos of cyanobacterial PORs and diatom POR1 and POR2 proteins.** Alignment of sequence logos of cyanobacterial POR proteins with diatom POR1 and POR2 proteins. Amino acid position indicated at the right of each line, corresponding to cyanobacterium *Synechocystis elongatus* and diatom *Phaeodactylum tricornutum*. Boxes indicate characteristic motifs, with diagnostic amino acids marked with asterisks: (a) Rossman fold essential to NADPH binding; (b) Y, K residues essential to enzyme-cofactor-substrate coordination and proton donation; (c) cysteine essential to catalysis. Amino acids are colored according to their chemical properties: green are polar (GSTYC); purple are neutral (QN), blue are positively charged (KRH); red are negatively charged (DE); and black are hydrophobic (AVLIPWFM).

To explore the evolution of *por* genes, a database was compiled from: (a) in-house amplification and sequencing of stramenopile *por* genes; (b) the recently sequenced genome of the haptophyte *Chrysochromulina tobin* (Hovde, Starkenburg and Cattolico, in prep.) and (c) publically available genomes, transcriptomes, and sequences. Because the POR protein is affiliated with the large, fairly conserved SDR protein family [47,48], e-values alone were not used to identify *por* genes. Instead all putative *por* sequences were screened for the presence of specific motifs encompassing particular lysine, tyrosine, and cysteine residues. These amino acids were experimentally shown to be essential to POR enzyme catalytic function in cyanobacteria and land plants (Figure 2; [49-53]). Analyses showed that these criteria eliminate homologs from cyanobacteria as well as chlorophytic and CASH algae that comprise strongly supported branches that cannot be placed within a POR phylogeny with statistical certainty. These protein clusters potentially represent closely-related SDR proteins that perform distinct, as-yet-undescribed functions [54]. The use of specific amino acid diagnostic characters also eliminates two *por* genes that were putatively identified in microarray-based studies of the *P. tricornutum* chlorophyll synthesis pathway ([42,43]; their *por3* and *por4*).

**Figure 3:**
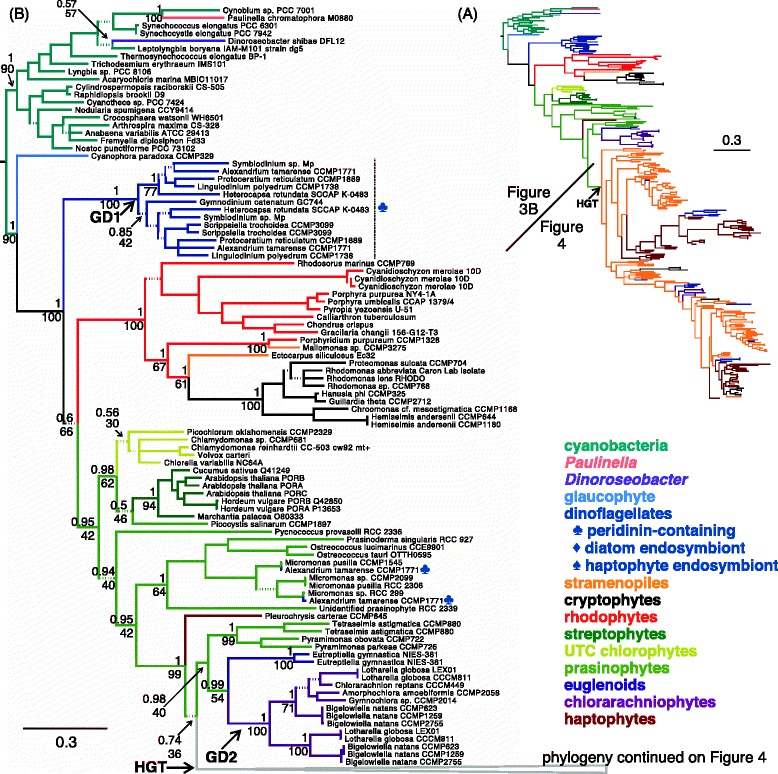
***Por*** **gene tree: rhodophytic identity of cryptophyte and some stramenopile** ***por*** **s; duplication of dinoflagellate and chlorarachniophyte** ***por*** **s. (A)** Outline of the full *por* gene tree inferred from the 274 amino acid conserved core of 275 POR proteins from cyanobacteria, eukaryotic algae and land plants, representing 162 taxa. Branches are colored according to algal lineage (see legend). The corresponding, detailed phylogeny is split between Figures 3B and 4. Scale bar indicates 0.3 amino acid substitutions per site. **(B)** Basal portion of *por* gene tree. Branches are colored according to algal lineage (see legend), with symbols indicating origin of endosymbiont in dinoflagellate taxa whose ancestral plastids have been replaced. Bayesian and maximum-likelihood analyses recovered nearly identical trees. Posterior probabilities are shown above branches and bootstrap support is shown below branches. All dashed branches have less than 0.95 posterior probability. Scale bar indicates 0.3 amino acid substitutions per site. Gene duplication (GD) and horizontal gene transfer (HGT) events are indicated with arrows.

**Figure 4:**
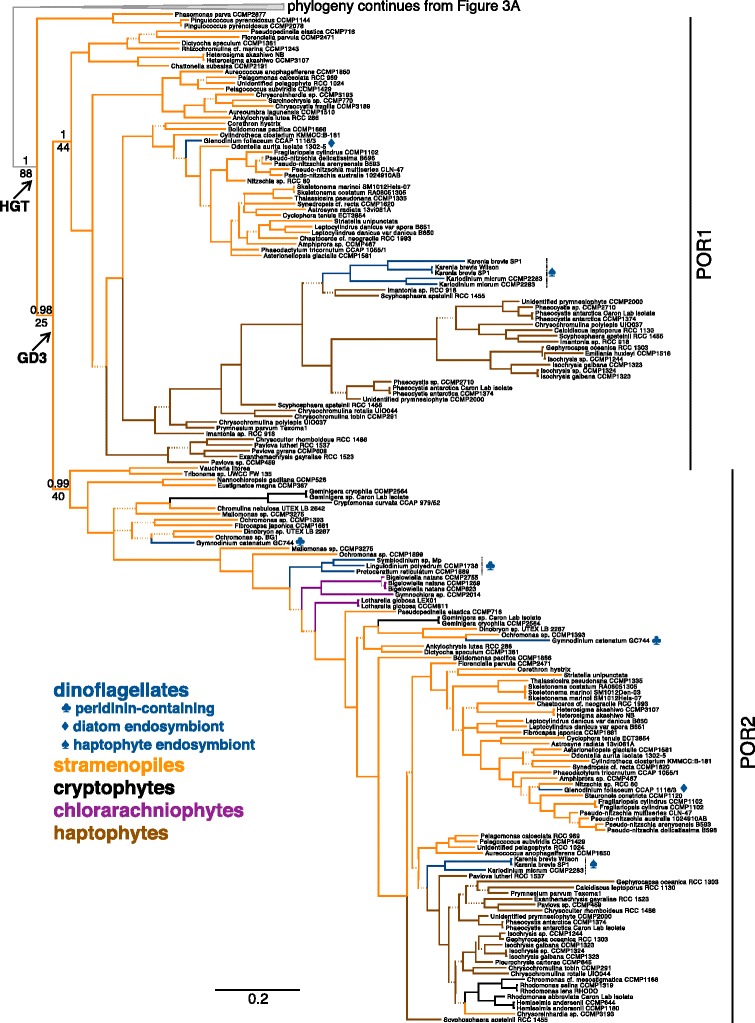
***Por*** **gene tree: duplication of xenologous stramenopile/haptophyte** ***por*** **genes.** Bottom half of the *por* gene tree outlined in Figure 3A. Branches colored by lineage, with symbols indicating origin of endosymbiont in dinoflagellate taxa whose ancestral plastid has been replaced (see legend). Posterior probabilities are shown above branches and bootstrap support are shown below branches. All dashed branches have less than 0.95 posterior probability. Scale bar indicates 0.3 amino acid substitutions per site. Arrows indicate the inferred horizontal gene transfer (HGT) of a *por* gene from prasinophytes to the stramenopiles, and the subsequent gene duplication (GD3) to create *por*1 and *por*2*.*

The resultant alignment of the 274 amino acid conserved core of 275 POR proteins from cyanobacteria, eukaryotic algae and land plants represents 162 taxa (Figure 3). The Whelan and Goldman matrix for globular proteins (WAG, [55]), a gamma shape parameter and a proportion of invariable sites were found to best fit the data and were therefore used in Bayesian and maximum-likelihood phylogenetic inference. Both methods returned nearly identical topologies. The entirety of the Bayesian phylogeny is shown in Figure 3A. Details of this gene tree are shown in Figures 3B and 4. Bayesian posterior probabilities and maximum-likelihood bootstrap values are indicated on all principal branches (Figures 3B and 4), with dashed branches indicating less than 0.95 posterior probability throughout the tree. The amino acid alignment and Bayesian and maximum-likelihood trees (with sequence accessions) are available in Additional files 1, 2, and 3. The identities of all sequences are tabulated in Additional file 4.

Given the cyanobacterial origin of the POR enzyme [3,47], POR proteins from this taxon were used to root the phylogeny shown in Figure 3. The POR protein phylogeny backbone follows the expected pattern based on current knowledge of the relationships among algal taxa originating from primary endosymbiosis. The *Paulinella chromatophora* POR clusters within the cyanobacterial outgroup, reflecting its close association with cyanobacteria as an alga derived from a unique primary endosymbiosis [56]. Among the Archaeplastida, the glaucophytic, rhodophytic and chlorophytic (including streptophyte, ulvophyte-trebouxiophyte-chlorophyte (UTC) clade, and prasinophyte) lineages branch deeply as expected [14,57], interrupted only by a branch of dinoflagellate POR proteins (discussed below). Our extensive POR protein phylogeny also confirms the cyanobacterial origin of the POR of *Dinoroseobacter shibae,* an anoxygenic phototroph believed to have obtained a *por* gene via horizontal transfer [58].

### Replacement of ancestral *por* gene with horizontally transferred *por* gene in stramenopile and haptophyte algae

Because the CASH algal lineages obtained their plastids from a rhodophytic source, the POR proteins of these lineages are expected to nest within the rhodophytic POR branch. As shown in Figure 3B, POR proteins of eight sampled cryptophyte species (*Chroomonas* cf. *mesostigmatica, Guillardia theta, Hanusia phi, Hemiselmis andersenii* [2 strains]*, Proteomonas sulcata, Rhodomonas abbreviata, Rhodomonas lens, Rhodomonas* sp.) and two stramenopiles (*Ectocarpus siliculosus* and *Mallomonas* sp.) demonstrate affinity with rhodophytic PORs. Previous studies identified a member of the Porphyridiales as the progenitor of all CASH plastids [59]. The fact that the phaeophycean, synurophycean, and cryptophyte POR proteins are sister to the *Porphyridium purpureum* POR protein suggests that the POR proteins found these taxa may represent the original rhodophyte-derived enzyme. We note, however, that the *por* genes of the two stramenopiles *E. siliculosus* and *Mallomonas* sp. are not sister to one another as would be expected given the shared origin of their plastids. Barring a complex scenario of two unique HGT events from the rhodophytes to the phaeophyceans and synurophyceans, the polyphyletic placement of these sequences may be due to insufficient phylogenetic signal.

In contrast, a chlorophytic POR protein origin is detected for most stramenopiles, all sampled haptophytes, several peridinin-containing dinoflagellates, and many cryptophytes. This relationship is indicated by the emergence of their proteins within the prasinophytic POR branches (indicated by arrows in Figures 3 and 4). These data suggest that the original rhodophytic *por* gene of these lineages has been replaced (or supplemented in some cases) by a prasinophytic *por* gene obtained by HGT. The loss of the ancestral rhodophytic *por* gene is further supported by analyses of the whole genome sequences of two haptophytes and five stramenopiles. Only *por* genes of chlorophytic origin are recovered from these complete genomes (*Emiliania huxleyii, Thalassiosira pseudonana*, *Phaeodactylum tricornutum, Fragilariopsis cylindrus, Pseudo-nitszchia multiseries* genomes: http://genome.jgi.doe.gov; *Nannochloropsis gaditana*: http://nannochloropsis.genomeprojectsolutions-databases.com; *Chrysochromulina tobin* genome: Hovde, Starkenburg and Cattolico, unpublished).

The presence of only two stramenopiles that exhibit a POR protein in the rhodophyte clade (Figure 3B) is enigmatic. It is unclear whether most phaeophyceans (e.g., *E. siliculosus*) or synurophyceans (e.g., *Mallomonas* sp.) retain a rhodophytic-type POR*.* These two stramenopile classes are not closely related to one another, but rather belong to the stramenopile SI and SII clades (of three total clades), respectively [60]. They are each more closely related to classes whose members appears to solely possess the prasinophytic *por* gene. Stramenopile classes in this study that possess *por* genes of prasinophytic origin include members of SI (Raphidophyceae and Xanthophyceae), SII (Chrysophyceae, Eustigmatophyceae and Pinguiophyceae), as well as the SIII (Bacillariophyceae, Bolidophyceae, Dictyochophyceae, and Pelagophyceae) clades. The presence of the prasinophytic *por* gene in all three stramenopiles clades, the derived position of the Phaeophyceae and Synurophyceae within the stramenopiles, and the presence of both rhodophytic *and* prasinophytic *por* genes in *Mallomonas* sp. suggest that the xenologous *por* gene was obtained by stramenopiles early in their evolution. Similarly, the absence of rhodophytic *por* genes in haptophytes and presence in each sampled species of the xenologous *por* gene suggests that the rhodophytic *por* gene of haptophytes was replaced early in their evolution.

The *por* gene identities of cryptophytes are highly variable. A rhodophytic *por* appears to be the only *por* in *Guillardia theta, Hanusia phi, Proteomonas sulcata*, and *Rhodomonas* sp. Cryptophytes bearing rhodophytic *por*s that also have *por*s related to the clade of xenologous stramenopile/haptophyte *por*s include *Chroomonas* cf. *mesostigmatica, Hemiselmis andersenii, Rhodomonas abbreviata,* and *Rhodomonas lens*. Lastly, *Cryptomonas curvata, Geminigera cryophila, Geminigera* sp., and *Rhodomonas salina* appear to have only the xenologous stramenopile/haptophyte *por*. However, it is possible that not all *por* genes were present in the sampled transcriptomes. Notably, within the branch of xenologous stramenopile *por* genes (Figure 4), stramenopile and haptophytic *por* genes generally cluster together. This association is not true for cryptophyte *por* genes, which are spread among three branches far apart from one another, suggesting that the xenologous stramenopile/haptophyte *por* genes of cryptophytes might originate from several independent HGTs or possibly represent phylogenetic artifacts or transcriptome contamination [61].

Similar to the cryptophytes, several peridinin-containing dinoflagellate species (*Gymnodinium catenatum, Symbiodinium* sp., *Lingulodinium polyedrum* and *Protoceratium reticulatum*) and several chlorarachniophytes (*Lotharella globosa*, *Gymnochlora* sp., *Bigelowiella natans*) possess a copy of the xenologous stramenopile/haptophyte *por*. The punctate nature of these POR protein identities within the phylogeny (Figure 4) suggests that the genes were obtained by HGT, though artifacts of poor phylogenetic signal or contamination must be considered. Extensive gene acquisition via HGT from a variety of sources has been documented to occur in dinoflagellates (e.g., [32,62-64]) and the chlorarachniophyte *B. natans* [36,65,66]. Given the propensity of dinoflagellates to obtain exotic genes, we also note that the peridinin-containing dinoflagellate *Alexandrium tamarense* appears to harbor two prasinophytic POR proteins, possibly obtained from a unique HGT event sourced from a *Micromonas*-like species (Figure 3B).

### Duplication of the xenologous stramenopile/haptophyte *por* gene

The branches of the POR protein phylogeny pertaining to the xenologous POR enzymes are shown in Figure 4. Multiple POR xenologs are recovered from many of the surveyed stramenopile and haptophyte species. In most cases, each of two gene copies is distributed between two principal branches in the tree—evidence that a gene duplication event has occurred. Whether this duplication event took place: (a) in the lineage that first obtained the xenolog (most parsimonious); (b) in an unsampled prasinophyte lineage prior to the HGT of both paralogs, or (c) resulted from a near simultaneous incorporation of two copies of the same gene, cannot be determined with certainty.

In Figure 4a, the node representing the gene duplication event is labeled as GD3, and resultant POR paralogs are annotated as POR1 (gene*: por*1) and POR2 (gene: *por2*). The maintenance of both POR1 and POR2 is fairly-well conserved: 16 of 22 haptophyte taxa and 34 of 56 stramenopile taxa possess both *por1* and *por2* genes. Those lacking the full *por1/por2* gene compliment possess solely one paralog or occasionally two of one paralog (see Additional file 4 for data in tabular format). Note that incomplete transcriptomic data or poor gene predictions in genomic datasets may obscure the identification of a second paralog for some species in this study.

One of two *Pleurochrysis carterae* (Haptophyta; Coccolithales) POR proteins, as well as the sole POR proteins of *Pinguiococcus* spp. and *Phaeomonas parva* (Stramenopila; Pinguiophyceae) branch before the duplication event shown in Figure 4. The placement of the *P. carterae por* within prasinophytic PORs (Figure 3) may simply be an artifact of phylogenetic uncertainty, since its placement changed as taxa were added to the phylogeny (data not shown). Alternatively, the *P. carterae por* could represent a unique HGT event from a prasinophyte to just this taxon. The second *P. carterae por* placed as expected within the stramenopile/haptophyte POR2 clade. The Pinguiophyceae are not expected to be sister to the rest of the stramenopiles [60,67], thus the placement of their PORs at the base of the duplication event is enigmatic. Support for the monophyly of the xenologous stramenopile/haptophyte PORs is high (posterior probability 1, bootstrap 88). The POR1 and POR2 branches are distinguished with high posterior support (1 and 0.99, respectively) but low bootstrap support (44 and 40, repectively). Given the subsampling algorithm used in bootstrapping, the low bootstrap support at these nodes likely reflects the fact that a small subset of amino acid positions are diagnostic for the stramenopile/haptophyte POR1 versus POR2 proteins, as shown in Figure 2 [68].

### Evolutionary significance of the xenologous stramenopile/haptophyte *por* genes

Researchers studying algae bearing plastids of rhodophytic origin frequently document the occurrence of nuclear-encoded genes of chlorophytic origin. For example, a phosphoribulokinase of chlorophytic origin was found in CASH algae [31], and Frommolt et al. [69] reported that five of the 16 carotenoid biosynthesis genes in cryptophyte, haptophyte, and stramenopile algae were also from chlorophytes. Recent meta-analyses of genomic data have reported the presence of many chlorophytic genes in diatoms (Stramenopila; [70]), a chromerid (Alveolata; [71]) and pico-prymnesiophytes (Haptophyta; [72]). Some researchers have attributed high levels of green genes in CASH lineages to putative cryptic endosymbiotic gene transfer (EGT) events (e.g., [69,70,72]), while others have invoked poor taxon sampling, a lack of manual curation, and phylogenetic error to explain these findings [71,73,74]. We note that, although the possibility for phylogenetic error is omnipresent, our study benefits from: (a) the inclusion POR protein sequences from many rhodophytic (including mesophilic), chlorophytic, and other algal taxa; (b) manual curation of sequence data and alignments; (c) special attention paid to support values at key nodes on the tree when making inferences about sequence origin; as well as (d) data exploration [e.g., in a POR protein phylogeny inferred without chlorophytic PORs, the stramenopile/haptophyte POR clade remained sister to rather than derived from rhodophytic PORs (data not shown)]. Furthermore, the horizontal transfer of at least some genes can be expected for phagocytotic algae (or algae with phagocytotic ancestors) [29]. Representatives within the cryptophytes, haptophytes, stramenopiles and dinoflagellates are commonly phagocytotic.

Whereas the prasinophytic origin of these xenologous POR proteins is unambiguous, the history of these xenologs among CASH taxa is less clear. Like chloroplast-encoded genes, nuclear-encoded chloroplast-targeted genes serve as markers for plastid origin because they are transferred from the symbiont to the host during endosymbiosis. However, HGT presents another potential route of transfer between lineages that can obscure relationships among groups.

As discussed above, the punctate nature of the xenologous *por* gene distribution in cryptophytes, dinoflagellates, and chlorarachniophytes suggests that these genes were obtained from several unique HGT events to these groups. In contrast, the xenologous *por* genes appear in all but one stramenopile and all haptophytes in our extensive sampling of 11 classes and all three clades of stramenopiles as well as six orders of haptophytes including the basal lineage Pavlovales [75]. The ubiquitous presence of the xenolog in stramenopiles and haptophytes suggests that this prasinophytic *por* was acquired early in the evolution of these taxa.

Fascinatingly, stramenopile *por*s are found ancestral to haptophyte *por*s in the phylogeny presented in Figure 4, especially those from members of the Pelagophyceae. Although statistical support for the exact placement of haptophyte PORs (and PORs of dinoflagellates with haptophyte-derived plastids) in the phylogeny is weak, stramenopile PORs occupy strongly supported basal nodes within both the POR1 and POR2 branches. The derived position of the haptophyte PORs suggests transfer of the xenologous *por* genes from stramenopiles to haptophytes. Under the aforementioned rhodoplex hypothesis, an endosymbiotic origin for the *por* xenolog duplicates of haptophytes would necessarily invoke plastid transfer from the stramenopiles to the haptophytes, likely after the stramenopile plastid lineage diverged from that of cryptophytes (which retain a relic nucleomorph unlike other CASH plastids [76]).

The relationship between the plastids of stramenopile and haptophytic algae is presently unresolved [18,25,26,77,78]. Using a BLAST-based statistical approach, Stiller and colleagues recently documented strong support for a model of serial endosymbiosis wherein the plastid was transferred from rhodophytes → cryptophytes → stramenopiles → haptophytes [79]. Furthermore, some plastid phylogenies find stramenopiles and haptophytes sister to one another to the exclusion of cryptophytes (e.g., [17,78]). Assuming serial endosymbiosis from stramenopiles to haptophytes, limited taxon sampling may explain why haptophytes were observed sister to rather than derived from the stramenopiles in these plastid phylogeny studies. The present study, although limited to just one gene, incorporates a diverse array of haptophytes and stramenopiles and may therefore be expected to better resolve such a relationship. Under this scenario, low support for the exact placement of haptophytic *por*s may be due to extinction of the stramenopile donor taxon.

In contrast to the above findings, other plastid phylogenies find cryptophytes and haptophytes more closely related to one another than they are to stramenopiles (e.g., [79-81]). Importantly, a shared, horizontally transferred *rpl36* gene encoded in the chloroplasts of only cryptophytes and haptophytes strongly indicates a sister relationship between these two taxa [82,83]. If haptophyte and cryptophyte plastids are indeed more closely related to one another than to stramenopile plastids, the xenologous *por* genes would have to have been transferred from stramenopiles to haptophytes via HGT early in the evolution of the haptophytes. However, just as a consensus concerning the relationships of CASH plastids has not yet been reached, the relative ages of the various CASH lineages remain unresolved [84-86].

### POR protein identity post-duplication

Stramenopile/haptophyte POR protein identity post-duplication is demonstrated in the sequence logos of Figure 2. Diatom POR1 and POR2 amino acid sequences were used to best represent these xenologous POR proteins without the many small gaps present in an alignment of all stramenopile/haptophyte PORs. High sequence conservation is shown when POR1 and POR2 are compared to ancestral, cyanobacterial PORs. The core region of diatom PORs (excluding signal and transit peptides and a C-terminal extension on diatom POR2) share 60% sequence similarity with cyanobacterial PORs. Each diatom POR appears to maintain conserved regions particular to that POR protein as well as to all POR proteins; sequence similarity is 75% within diatom POR1, whereas sequence similarity is lower at 66% within diatom POR2, principally due to a poorly conserved C-terminal extension. This C-terminal extension results in a predicted protein of ~60kD rather than the typical ~40kD (Figure 2, [87]). Sequencing the *Phaeodactylum por2* cDNA amplified by 3′ RACE shows that this extension is transcribed (Hunsperger and Cattolico, unpub.). Notably, antibodies raised against heterologously expressed, full-length *Phaeodactylum* POR2 proteins show cross reactivity to a 40kD product in *Phaeodactylum* cell extracts. This observation suggests that POR2 is post-transcriptionally truncated to a conventional POR2 size (Hunsperger and Cattolico, unpub.). Thus both proteins are expected to be functional.

### Identity of *por* genes in dinoflagellates with haptophyte and diatom endosymbionts

The POR proteins of dinoflagellates whose plastids have been replaced by those of a haptophyte (*Karenia brevis, Karlodinium micrum*) or diatom (*Glenodinium foliaceum*, a “dinotom”) appear to originate from the haptophyte or diatom endosymbiont, respectively (Figure 4). Just as diatoms and haptophytes each have two unique POR proteins, dinoflagellates that bear plastids originating from these algal sources also possess these same two unique POR proteins. Given that the haptophyte and diatom plastids replaced the ancestral peridinin-containing plastids, it is expected that ancestral POR proteins were already integrated into the dinoflagellate nuclear genome. These ancestral POR proteins were lost, however, rather than re-targeted to the new chloroplast. One might speculate that regulatory or functional schemes unique to each of the new endosymbiont’s two *por* genes favored the retention of these new *por* genes. It would be interesting to determine whether this *por* gene substitution pattern also holds for dinoflagellates bearing chlorophyte (e.g., *Lepidodinium*; [88]) or ephemeral cryptophyte (e.g., *Dinophysis;* [89]) derived plastids. Whereas haptophytic endosymbionts no longer possess nuclei, identifiable nuclei remain in diatom endosymbionts [90]. As the dinotom *por* genes used in this study were obtained from transcriptomes, it is unclear whether they are encoded within the endosymbiont’s nucleus or have been transferred to the dinoflagellate nucleus. Nonetheless, the diverse origins of dinoflagellate POR proteins reflect the propensity of members of this taxon for foreign gene acquisition, endosymbiont replacement and genetic remodeling (e.g., [32,62-64,90,91]).

### Additional *por* gene duplications

#### Duplicated dinoflagellate-specific por genes

Because chloroplasts of ancestral, peridinin-containing dinoflagellates have been shown to be of rhodophytic origin [20,59,92], the *por* genes of dinoflagellates can be expected to group within the rhodophytes. Instead, a dinoflagellate-specific group of POR proteins is found sister to rhodophytic and chlorophytic algae, indicating an unresolved origin for this unique group of enzymes (Figure 3).

The recurrence of five dinoflagellate taxa in each of the two main branches of the dinoflagellate POR subtree is classic evidence of a gene duplication event (annotated in Figure 3A as GD1). Low support values for one of these branches, however, makes the nature of the gene duplication less clear. Because all seven taxa in these branches utilize peridinin, which is thought to be the ancestral photosynthetic dinoflagellate pigment [90], these paralogous POR proteins of uncertain origin may have been acquired early in the evolution of the dinoflagellates.

#### Duplication of chlorarachniophyte and euglenoid por genes

The euglenids and chlorarachniophytes are algal lineages originating from two separate secondary endosymbioses involving chlorophytic algae. The euglenid chloroplast originates from the Pyramimonadales lineage of the prasinophyte algae, whereas the chlorarachniophytes engulfed an alga of uncertain identity from the UTC clade [17,88,93,94]. Phylogenetically, the POR proteins of a euglenid and several chlorarachniophytes are found sister to one another and nested within prasinophyte algae closest to a branch containing pyramimonads (*Pyramimonas* spp.) and a chlorodendrophyte (*Tetraselmis astigmatica*). The placement of the euglenid PORs is broadly congruent with the known origin of their plastids from pyramimonads. We note that the sole Euglenoid included in these studies, *Eutreptiella gymnastica*, appears to possess two *por* genes, perhaps indicating that a *por* gene duplication event occurred in this taxon.

A sister relationship between the euglenids and chlorarachniophytes, however, is inconsistent with the separate origins of the plastids of these two groups. Improper placement of the chlorarachniophyte POR proteins may be due to the inclusion of very few UTC chlorophyte species and few euglenids in the POR protein tree. Alternatively, POR placement could reflect a horizontal gene transfer from the prasinophytes to the chlorarachniophytes, though additional taxon sampling would be necessary to verify such an event.

These chlorarachniophyte-specific POR proteins show evidence of gene duplication (labeled GD2 in Figure 3B). Three strains of *Bigelowiella natans* and two strains of *Lotharella globosa* appear to each have two chlorarachniophyte-specific POR proteins that are divided between the two main branches in this clade. The basal position of the split between the two POR paralogs supports a gene duplication event in the common ancestor of most chlorarachniophytes, given that *Amorphochlora amoebeformis* possesses one of the paralogs and represents an early diverging branch of chlorarachniophytes [95]. It is unclear whether *Amorphochlora amoebeformis, Gymnochlora* sp., and *Chlorarachnion reptans* then lost one paralog, or whether incomplete transcriptomic data impeded the recovery of the second paralog.

### Physiological significance of multiple *por* genes

The discovery of multiple *por* genes in a species is not without precedent. Although some species of Viridiplantae are confirmed to have just one *por* gene, numerous representatives within this taxon encode multiple *por* genes (reviewed in [47]). Phylogenetic analysis suggests that some of the angiosperm POR paralogs are shared among select plant species, while other paralogs arose more recently and are unique to a particular taxon. In vascular plants, light and developmental stage appears to regulate the expression of each *por* gene. For example, in the angiosperm *Arabidopsis thaliana,* two POR isoenzymes, PORA and PORB, accumulate in dark-adapted seedlings in concert with increasing levels of the pigment substrate Pchlide. As a result, the plant is poised for chlorophyll synthesis upon illumination of the seedling. PORA is quickly degraded upon seedling exposure to light, while PORB continues to be expressed in mature tissues and thus is primarily responsible for continued chlorophyll production. A third POR, PORC, is up-regulated with increasing light intensity, enabling higher chlorophyll abundances under high light (reviewed in [47,87]).

Given intrinsic differences between the life histories, physiologies and ecologies of lands plants and algae, it will be interesting to compare their regulatory and functional schemes for chlorophyll biosynthesis. One might anticipate that, similar to land plants, the regulation of multiple *por* homologs in algae may be tied to light availability (varying with time of day, season, water turbidity and depth) and developmental stage (e.g., encystment/excystment). For example, whereas many land plants increase their chlorophyll levels in response to high light [96], algal chlorophyll levels *decrease* as light intensity increases [97]. As expected, the transcription of both *Phaeodactylum tricornutum por1* and *por2* were found to be initially down-regulated in response to a transition from low (35 μM photons m^-2^ s^-1^) to high (500 μM photons m^-2^ s^-1^) light levels [42]. Our own RT-qPCR measurements of *P. tricornutum por1* and *por2* mRNA abundance shows a unique oscillatory pattern for each gene over a 12 hour light:12 hour dark photoperiod (Hunsperger and Cattolico, in prep), suggesting independent regulation of these two genes. Similarly, transcriptomic analysis of 12 hour light:12 hour dark synchronized *Chrysochromulina tobin* (Haptophyta; Prymnesiales) cultures indicates that the two *por* genes independently respond to the imposed light/dark cues in a pattern that differs from that seen for *P. tricornutum por1* and *por2* (Hovde and Cattolico, unpub).

### Loss of chloroplastic genes encoding LIPOR

At least one *por* gene, encoding the light-dependent protochlorophyllide oxidoreductase (POR), has been documented in all sequenced algal nuclear genomes. In contrast, chloroplast genome sequencing has revealed the loss or degradation of the three chloroplast-localized genes encoding the light-independent protochlorophyllide oxidoreductase (LIPOR; *chlL, chlN,* and *chlB*) in members of the chlorophytic, euglenoid and chlorarachniophyte algae (Table 1) as well as rhodophytic and CASH algae (Table 2) (see also [4,45,46,80,94]). Furthermore, the loss of these genes is well documented for angiosperms (reviewed in [4]).

**Table 1:** **Distribution of** ***por*** **genes and chloroplast-encoded LIPOR genes in chlorophytic algae, chlorarachniophytes, and euglenids**

			***Por*** **genes in genus (this paper)**	**Chloroplast-encoded LIPOR genes**			**Chloroplast genome accession**
**Taxon**	**Species**	**Culture ID**		***chlL***	***chlN***	***chlB***	
**Chlorophyta**							
Chlorophyceae	*Acutodesmus obliquus*	UTEX 393		+	+	+	NC_008101
Chlorophyceae	*Chlamydomonas reinhardtii*	n/a	1	+	+	+	NC_005353
Chlorophyceae	*Dunaliella salina*	CCAP 19/18		+	+	+	NC_016732
Chlorophyceae	*Floydiella terrestris*	UTEX 1709		+	+	+	NC_014346
Chlorophyceae	*Gonium pectorale*	K3-F3-4		+	+	+	NC_020438
Chlorophyceae	*Oedogonium cardiacum*	SAG 575-1b		+	+	+	NC_011031
Chlorophyceae	*Pleodorina starrii*	NIES 1363		+	+	+	NC_021109
Chlorophyceae	*Schizomeris leibleinii*	UTEX LB 1228		+	+	+	NC_015645
Chlorophyceae	*Stigeoclonium helveticum*	UTEX 441		+	+	+	NC_008372
Mamiellophyceae	*Micromonas pusilla*	RCC299	1	-	-	-	NC_012575
Mamiellophyceae	*Monomastix sp.*	OKE-1		-	-	-	NC_012101
Mamiellophyceae	*Ostreococcus tauri*	OTTH0595	1	-	-	-	NC_008289
Nephroselmidophyceae	*Nephroselmis olivacea*	NIES 484		+,2	+,2	+,2	NC_000927
Prasinophyceae	*Pycnococcus provasolii*	CCMP1203	1	+	+	-	NC_012097
Prasinophyceae	*Pyramimonas parkeae*	CCMP726	1	+,2	+,2	+	NC_012099
Trebouxiophyceae	*Chlorella variabilis*	NC64A	1	+	+	+	NC_015359
Trebouxiophyceae	*Chlorella vulgaris*	C-27	1	+	+	+	NC_001865
Trebouxiophyceae	*Coccomyxa subellipsoidea*	C-169		+	+	+	NC_015084
Trebouxiophyceae	*Leptosira terrestris*	UTEX 333		+	+	+	NC_009681
Trebouxiophyceae	*Parachlorella kessleri*	SAG 211/11 g		+	+	+	NC_012978
Trebouxiophyceae	*Pedinomonas minor*	UTEX LB 1350		-	-	-	NC_016733
Trebouxiophyceae	*Trebouxiophyceae sp.*	MX-AZ01		+	+	+	NC_018569
Ulvophyceae	*Bryopsis hypnoides*	n/a		+	+	+	NC_013359
Ulvophyceae	*Oltmannsiellopsis viridis*	NIES 360		+	+	+	NC_008099
Ulvophyceae	*Pseudendoclonium akinetum*	UTEX 1912		-	-	-	NC_008114
**Cercozoa**							
Chlorarachniophyceae	*Bigelowiella natans*	CCMP621	3	-	-	-	NC_008408
Chlorarachniophyceae	*Lotharella oceanica*	CCMP622	3	-	-	-	KF438023
**Euglenozoa**							
Euglenophyceae	*Euglena gracilis*	Z		-	-	-	NC_001603
Euglenophyceae	*Euglena viridis*	ATCC PRA-110		-	-	-	NC_020460
Euglenophyceae	*Eutreptiella gymnastica*	K-0333	2	-	-	-	NC_017754
Euglenophyceae	*Monomorphina aenigmatica*	UTEX 1284		-	-	-	NC_020018

(+) Present in chloroplast genome; (**-**) not present in fully-sequenced chloroplast genome. The number of *por* genes found in this study for a particular genus (not necessarily the same species or strain) is also indicated.

**Table 2:** **Distribution of** ***por*** **genes and chloroplast-encoded LIPOR genes in rhodophytes and CASH algae**

			***Por*** **genes in genus (this paper)**	**Chloroplast-encoded LIPOR genes**			**Chloroplast genome accession**
**Taxon**	**Species**	**Culture ID**		***chlL***	***chlN***	***chlB***	
**Rhodophyta**							
Bangiophyceae	*Cyanidioschyzon merolae*	Strain 10D	3	-	-	-	NC_004799
Bangiophyceae	*Cyanidium caldarium*	RK1		-	-^Δ^	-	NC_001840
Bangiophyceae	*Porphyra purpurea*	Avonport	1	+	+	+	NC_000925
Bangiophyceae	*Pyropia haitanensis*	PH-38 (voucher)	1	+	+	+	NC_021189
Bangiophyceae	*Pyropia yezoensis*	U-51	1	+	+	+	NC_007932
Florideophyceae	*Calliarthron tuberculosum*		1	+	+	+	NC_021075
Florideophyceae	*Chondrus crispus*		1	-	-	-	NC_020795
Florideophyceae	*Gracilaria tenuistipitata var. liui*		1	-	-	-	NC_006137
Florideophyceae	*Gracilaria salicornia*	ARS08332 (voucher)	1	-	-	-	KF861575
Halymeniaceae	*Grateloupia taiwanensis*			-	-	-	NC_021618
Porphyridiophyceae	*Porphyridium purpureum*	NIES 2140	1	-	-	-	AP012987
**Cryptophyta**							
Chroomonadaceae	*Chroomonas mesostigmatica*	CCMP1168	2	+	ψ	?	EU233753; EU233756
Chroomonadaceae	*Chroomonas pauciplastida*	CCMP268	2	+	+	+	EU233754; EU233755; EU233748
Chroomonadaceae	*Hemiselmis andersenii*	CCMP644	2	+	+	+	EU233749; EU233750; EU233747
Chroomonadaceae	*Hemiselmis tepida*	CCMP443	2	+	+	?	EU233751; EU233752
Geminigeraceae	*Guillardia theta*		1	-	-	-	NC_000926
Pyrenomonadaceae	*Rhodomonas salina*	CCMP1319	1–2	ψ	ψ	ψ	NC_009573
**Haptophyta**							
Isochrysidales	*Emiliania huxleyi*	CCMP373	1	-	-	-	NC_007288
Pavlovales	*Pavlova lutheri*	ATCC 50092	1–2	-	-	-	NC_020371
Phaeocystales	*Phaeocystis antarctica*	CCMP1374	2–3	-	-	-	NC_016703
Phaeocystales	*Phaeocystis globosa*	Pg-G(A)	2–3	-	-	-	NC_021637
Prymnesiales	*Chrysochromulina tobin*	CCMP291	2	-	-	-	KJ201907
**Stramenopila**							
Bacillariophyceae	*Fistulifera sp.*	JPCC DA0580		-	-	-	NC_015403
Bacillariophyceae	*Odontella sinensis*		2	-	-	-	NC_001713
Bacillariophyceae	*Phaeodactylum tricornutum*	CCAP1055/1	2	-	-	-	NC_008588
Bacillariophyceae	*Synedra acus*			-	-	-	NC_016731
Bacillariophyceae	*Thalassiosira oceanica*	CCMP 1005	2	-	-	-	NC_014808
Bacillariophyceae	*Thalassiosira pseudonana*	CCMP 1335	2	-	-	-	NC_008589
Dictyochophyceae	*Apedinella radians*	CCMP1767		-	-	-	unpublished data*
Dictyochophyceae	*Rhizochromulina marina*	CCAP950/1	1	+	+	+	unpublished data*
Eustimatophyceae	*Nannochloropsis gaditana*	CCMP526	1	+	+	+	KJ410682
Eustimatophyceae	*Nannochloropsis oceanica*	LAMB0001	1	+	+	+	KJ410683
Eustimatophyceae	*Nannochloropsis oculata*	CCMP525	1	+	+	+	KJ410684
Eustimatophyceae	*Nannochloropsis salina*	CCMP1776	1	+	+	+	KJ410685
Pelagophyceae	*Aureococcus anophagefferens*	CCMP1984	2	-	-	-	NC_012898
Pelagophyceae	*Aureoumbra lagunensis*	CCMP1507	1	+	+	+	NC_012903
Pelagophyceae	*Pelagomonas calceolata*	CCMP1756	2	-	-	-	unpublished data*
Phaeophyceae	*Desmarestia aculeata*	KU-1141		+	+	?	unpublished data*
Phaeophyceae	*Ectocarpus siliculosus*	Ec32 (CCAP1310/4)	1	+	+	+	NC_013498
Phaeophyceae	*Fucus vesiculosus*			+	+	+	NC_016735
Phaeophyceae	*Nereocystis lutkeana*	UWCC MA 708		+	+	+	unpublished data*
Phaeophyceae	*Saccharina japonica*			+	+	+	NC_018523
Pinguiophyceae	*Pinguiococcus pyrenoidosus*	CCMP2188	1	+	+	+	unpublished data*
Raphidophyceae	*Chattonella subsalsa*	CCMP217	1	+	+	+	unpublished data*
Raphidophyceae	*Heterosigma akashiwo*	CCMP452	2	-	-	-	EU168191
Raphidophyceae	*Heterosigma akashiwo*	NIES293	2	-	-	-	NC_010772
Synurophyceae	*Synura petersenii*	CCMP854		-	-	-	unpublished data*
Xanthophyceae	*Botrydium cytosum*	UTEX 157		+	+	+	unpublished data*
Xanthophyceae	*Tribonema aequale*	CCMP1275	1	+	+	+	unpublished data*
Xanthophyceae	*Vaucheria litorea*	CCMP2940	1	+	+	+	NC_011600
**Dinophyta**							
Dinotrichales (dinotom)	*Durinskia baltica*	CS-38		-	-	-	NC_014287
Dinotrichales (dinotom)	*Kryptoperidinium foliaceum*	CCMP1326		-	-	-	NC_014267
**Chromerida**							
Chromeraceae	*Chromera velia*	CCMP2878		-	-	-	NC_014340
Vitrellaceae	*Vitrella brassicaformis*	CCMP3155/RM11		+, 2	+, 2	+	NC_014345

(+) Present in chloroplast genome or Fong and Archibald [46] study; (-) not present in fully-sequenced chloroplast genome; (ψ) present as pseudogene; (?) unknown; (*) Cattolico, Rocap and McKay; (^Δ^) *C. caldarium* re-annotated as per [6]. The number of *por* genes found in this study for a particular genus (not necessarily the same species or strain) is also indicated.

Typically, the three LIPOR genes are either entirely present or completely absent from a chloroplast genome. Notably, both sampled chlorarachniophytes, all four euglenoids, and all five haptophytes lack LIPOR genes in their chloroplasts, suggesting that LIPOR gene loss may have occurred early in the establishment of these lineages. In contrast, species with and species without chloroplastic LIPOR genes are documented for the chlorophytes, rhodophytes, cryptophytes, heterokonts, and chromerids. Such heterogeneity is seen even at the level of taxonomic class, with some members maintaining and other members having lost LIPOR genes in the Trebouxiophyceae, Ulvophyceae and Prasinophyceae (Chlorophyta), Bangiophyceae and Florideophyceae (Rhodophyta), as well as the Dictyochophyceae, Pelagophyceae, and Raphidophyceae (Stramenopila). The prasinophycean *Pycnococcus provasoli* appears to have lost solely the *chlB* gene and some cryptophytes are documented to possess LIPOR pseudogenes, showcasing the gradual process of LIPOR gene loss ([46,94]; Table 2).

These data from chloroplast genomes do not exclude the possibility that the three genes encoding LIPOR have, in some species, been moved to the nuclear genome via endosymbiotic gene transfer. BLASTp searches of all accessible, completely sequenced algal nuclear genomes for which chloroplastic *chlL*, *chlN*, or *chlB* genes are absent did not reveal nuclear homologs to these three genes (chlorophytes *Micromonas pusilla* and *Ostreococcus tauri;* cryptophyte *Guillardia theta*; stramenopiles *Aureococcus anophagefferens, Fragilariopsis cylindrus*, *Phaeodactylum tricornutum, Thalassiosira pseudonana;* haptophytes *Chrysochromulina tobin* and *Emiliania huxleyi*).

Bayesian and maximum-likelihood phylogenetic analysis of each LIPOR gene was also performed. Adding the genes in Tables 1 and 2 to the expansive survey of Sousa et al. [54], homologs were sampled from extant phyla known to possess *chlL, chlN and chlB*: those in Tables 1 and 2, cyanobacteria, chlorobacteria, chloroflexi, proteobacteria, firmicutes and acidobacteria. Similar to previous findings [46,54], the LIPOR genes of eukaryotic algae and a particular subset of cyanobacteria formed a monophyletic group. Resolution among phyla was correlated with protein length, with phyla well resolved only by the longest protein, CHLB (404 amino acids in alignment). Convincing evidence of horizontal transfer of any LIPOR gene was not detected for any eukaryotic alga (data not shown).

### Maintenance of non-homologous, isofunctional enzymes

*Why are* por *genes seemingly ubiquitous in plant and algal genomes, whereas LIPOR genes are lost in some lineages?* It has been suggested that oxygenic photosynthesis and present-day atmospheric oxygen levels are incompatibile with the oxygen-sensitive LIPOR enzyme [4,11,98]. Studies utilizing the cyanobacterium *Leptolyngbia boryana* (formerly *Plectonema boryanum*) and a *L. boryana por* knockout mutant were performed to probe this enzymatic constraint. Both the wild-type (encoding both POR and LIPOR proteins) and *por* knockout mutant grew equally well under low light intensities (10–25 μmol photons m^-2^ s^-1^). However, the mutant showed depressed growth and chlorophyll synthesis at medium light intensities (85 μmol photons m^-2^ s^-1^). At high light intensities (130 μmol photons m^-2^ s^-1^), the *por* knockout mutant failed to grow whereas the wild-type flourished ([99]). An increased rate of photosynthesis at high light intensities causes increased oxygen production that could impede LIPOR enzyme function. In support of this reasoning, later research determined that the *por* knockout mutant could grow when oxygen was continuously removed from the growth medium, although at only two-thirds the rate of the wild-type [8]. *In vitro* studies have identified the iron-sulfur clusters of LIPOR L-proteins as the primary targets of molecular oxygen [7,9]. The iron-sulfur cluster of the NB-proteins are much less vulnerable to oxygen [5,100,101].

Furthermore, the synthesis of an iron-requiring protein such as LIPOR may prove metabolically disadvantageous to phytoplankton living in iron-depleted regions such as the high-nutrient, low-chlorophyll regions of the subarctic and equatorial Pacific Ocean as well as the Southern Ocean [102,103]. Iron deficiencies have been shown to trigger a reduction in the synthesis of iron-rich proteins [104] and induce the substitution of functionally similar proteins that do not rely on this element, such as the replacement of ferredoxin by flavodoxin under iron-limiting conditions [105,106]. Future studies might determine whether low-iron conditions favor a switch between LIPOR and POR synthesis in algae possessing both enzymes.

*Why have some lineages maintained LIPOR genes?* Although the POR enzyme neither possesses iron moieties nor is sensitive to oxygen, light quantity and quality may affect the catalytic capacity of this enzyme. Studies in land plants have long identified that the absorption of light energy by Pchlide enables POR to catalyze its conversion [107]. The Pchlide pigment has absorbance maxima in both red and blue regions of the light spectrum [108]. Recently, Hanf et al. [109] showed that the photoconversion of Pchlide to Chlide by the POR enzyme was three to seven times as efficient when Pchlide absorbed red light (647 nm) rather than blue light (407 nm; though their choice of blue excitation wavelength for this experiment was controversial [110]). Due to its long wavelengths and concomitant lower energy, red light is attenuated rapidly from the water column, whereas green and especially blue light penetrates deeper. It is therefore possible that in deep or turbid waters or during an algal bloom, the POR enzyme may not efficiently enable chlorophyll production whereas the enzymatic ability of the LIPOR enzyme would not be expected to decrease under these conditions. In addition to enabling greening in the dark and low light, LIPOR would then also enable greening under red-light limited conditions. Future physiological experiments are warranted to explore whether a wavelength bias of the POR enzyme exists and, if so, to determine whether LIPOR provides a compensatory advantage.

*Could a* por *gene duplication compensate for a loss of LIPOR genes?* Interestingly, Tables 1 and 2 document a potential association between the loss of genes encoding LIPOR and the presence of duplicated *por* genes in both the haptophytes and stramenopiles (Tables 1 and 2). All five sequenced chloroplast genomes of haptophytes lack LIPOR genes, and 20 out of 22 sampled haptophytes maintain multiple *por* genes (stramenopile/haptophyte *por*1 and *por*2 genes, occasionally multiples copies of one paralog; Additional file 4). In those stramenopiles for which LIPOR and *por* gene complements are known, those species that lack LIPOR genes maintain multiple *por* genes (one stramenopile/haptophyte *por*1 gene and one stramenopile/haptophyte *por*2 gene; Additional file 4). The pattern of duplicated *por* genes in the absence of the isofunctional LIPOR enzyme is maintained even within taxonomic class. Within the Pelagophyceae, *Aureococcus anophagefferens* and *Pelagomonas calceolata* both lack LIPOR and each possesses two *por*s, whereas *Aureoumbra lagunensis* possesses LIPOR genes and maintains just one *por* gene. Similarly, within the Raphidophyceae, *Heterosigma akashiwo* lacks LIPOR but maintains two *por*s whereas *Chattonella subsalsa* maintains LIPOR genes and possesses just one *por* gene. The chlorarachniophyte *Bigelowiella natans* and the euglenid *Eutreptiella gymnastica* lack LIPOR genes, and both maintain multiple *por* genes. In contrast, two chlorophytes (*Micromonas pusilla* and *Ostreococcus tauri*), four rhodophytes (*Chondrus crispus*, *Gracilaria tenuistipitata* and *salicornia,* and *Grateloupia taiwanensis*) and two cryptophytes (*Guillardia theta* and some *Rhodomonas* spp.) lack LIPOR genes but possess just one *por* gene. A *por* gene duplication has not been documented, however, in these three taxa. A possible relationship between the maintenance of *por* gene duplicates and the loss of the LIPOR enzyme should be clarified as more algal genomes are sequenced.

Given that chlorophyll is only used in the light, the forestalling of chlorophyll synthesis due to the light-dependency of the POR enzyme may not prove problematic. For example, as in the etiolated seedlings of angiosperms discussed above, a dark-adapted alga that lacks LIPOR might accumulate POR enzymes complexed with Pchlide substrate and therefore be poised to produce large quantities of chlorophyll upon illumination. Our preliminary data also suggests that the capacity to differentially regulate *por* genes may be critical to algal cells as they progress through an alternate life history phase where light plays a seminal role. In transcriptomes developed from samples of the harmful-bloom forming alga *Heterosigma akashiwo* (Stramenopila; Raphidophyceae) which lacks LIPOR genes, *por*1 transcript abundance predominates in light-grown vegetative cells, whereas *por*2 appears to be highly up-regulated when resting phase cells are maintained in the cold and dark. Though hypothetical, these data suggest that stockpiling POR2 proteins may enable rapid chlorophyll synthesis upon the re-activation of resting cells initiated by light ([111,112]; Deodato and Cattolico, unpub.). These preliminary data merit rigorous study to determine if algae lacking LIPOR genes but possessing multiple *por* genes utilize one *por* gene copy to enable swift chlorophyll production upon a return to light.

## Conclusions

This study identifies conserved *por* gene duplications in: (a) dinoflagellates, (b) chlorarachniophytes, as well as (c) stramenopiles and haptophytes. These three *por* gene expansions offer a unique opportunity to study whether and how expanded gene sets with independent origins converge on similar regulatory schemes among evolutionarily divergent taxa. Even within the shared stramenopile and haptophyte *por* gene family, the ancient divergence of these two taxa may mean that they use their *por* gene sets differently—especially for those stramenopiles maintaining the LIPOR enzyme rather than multiple *por* genes. Given the loss of LIPOR genes from many species in various taxa, future studies are also warranted to clarify possible advantages of maintaining the LIPOR enzyme and whether iron limitation affects LIPOR synthesis.

The *por* gene duplicates of stramenopiles and haptophytes appear to arise from a horizontal gene transfer from a prasinophytic (chlorophytic) alga early in the evolution of the stramenopiles. The derived position of even basal haptophytes in comparison to stramenopiles evidences a possible gene transfer from the stramenopiles to the haptophytes, whether via EGT or HGT. Our data suggest that a thorough phylogenetic examination of chloroplast-targeted genes originally existing as single copies and shared among CASH lineages (e.g., *por*) may be a boon to the determination of CASH plastid relationships. The recent surge of publically available genomic and transcriptomic datasets should be mined for such informative genes [61].

## Methods

### *por* gene recovery

The following sources were used to retrieve POR genes for use in phylogenetic studies: (a) *public and private datasets*: *por* genes were identified by blast searching against public databases; in-house databases compiled from publically available genomes and transcriptomes, as well as the *Chrysochromulina tobin* CCMP291_RAC_ genome (Additional file 4). Transcriptomes reported to be derived from co-cultures (e.g., predator-prey experiments) were excluded from the analyses, although bacterized cultures were permitted because the *por* gene is not expected in non-photosynthetic organisms. (b) *algal samples:* Genomic DNA was extracted from algal cell pellets using Genomic-tip 500/G and 100/G DNA extraction kits (Qiagen, Valencia, CA) and targeted genes were recovered by PCR amplification and sequencing (Additional file 5). Degenerate primers were designed to universally amplify *por* sequence from diverse algal taxa (Additional file 5). Conserved protein regions for primer design were identified by aligning POR proteins from diverse algal taxa with the MUSCLE sequence alignment software (Edgar 2004). Degenerate primers were flanked with 23 bp of additional, non-degenerate nucleotides for ease of sequencing. POR genes were amplified in 25 μL reactions containing 0.1U/μL Lamda Biotech Tsg Plus DNA Polymerase (St. Louis, MO), 1X Tsg Plus reaction buffer, 0.2 mM dNTPs, 1.25 mM MgCl_2_, 1 ng/μL gDNA, and 1.2 μM each primer, with the addition of CES PCR additive when amplification proved problematic (described in Ralser et al. [113]). Cycling reactions were performed in an Eppendorf Mastercycler gradient thermocycler as follows: initial denaturation was at 94°C for 4 min; followed by 40 cycles of 30s denaturation at 94°C; 30s annealing at 50°C–58°C (gradient); a 2 min extension at 72°C; then 10 min final elongation at 72°C. When the only successful gene amplification for a given species occurred with an internal degenerate primer (i.e., not the degenerate primers closest to the 5′ or 3′ ends of the gene), a species-specific primer was designed ~200 bp from the appropriate sequence end and PCR was repeated with this new primer and the degenerate primer closest to the desired gene end.

When multiple bands or primer dimers were present in a PCR product, the desired band was gel extracted from a 1% agar Tris-Acetate-EDTA (TAE) gel stained with ethidium bromide. Gel extraction was performed using the QIAquick gel extraction kit (Qiagen). When sequencing yielded multiple products, the gene was re-amplified and extracted from a TAE gel stained with SeqJack GreenGene nucleic acid stain as per manufacturer’s recommendations (Mt. Baker Bio, Everett, WA) and visualized with blue light rather than UV light to retain DNA integrity. The extracted PCR product was cloned for re-sequencing using the TOPO TA cloning kit following manufacturer’s directions (Invitrogen, Carlsbad, CA). All sequencing was performed on an ABI 3130xl Genetic Analyzer using the ABI BigDye Terminator v3.1 Cycle Sequencing kit with 1/8th the manufacturer’s recommended reaction size (Applied BioSystems, Inc., Foster City, CA). pGEX primers (flanking the degenerate primer), species-specific internal primers, or M13 primers (cloned products) were used in the sequencing reactions.

When necessary, cDNA sequences were deduced from intron-containing gene sequences using GenomeScan [114-116], or by alignment with known POR protein sequences.

### POR protein curation

BLASTp searches for POR proteins returned many homologs, likely reflecting their origins in the conserved SDR (short-chain dehydrogenase-reductase) protein family [47,48]. Mutagenic studies of cyanobacterial and plant *por* genes have revealed several essential features of POR proteins: (a) the N-terminal Rossman fold (Gly-X-X-X-Gly-X-GLY) that is essential to NADPH binding ([117]; Figure 2a), (b) the Try-X-X-X-Lys array that stabilizes the enzyme-cofactor-substrate complex and whose Tyr donates a proton to Pchlide during the enzymatic reaction ([49-52]; Figure 2b), as well as (c) the cysteine residue determined to be essential to POR enzyme catalysis by Menon et al. ([53]; Figure 2c). Putative POR proteins were aligned with MUSCLE [118] and omitted if they lacked these diagnostic motifs. Sequences missing their N-termini (e.g., transcriptomic sequences) were not eliminated for lacking the N-terminal Rossman motif. Duplicate, short, and low-quality transcriptomic sequences (those with many undetermined amino acids) were removed.

### Phylogenetic inference

Curated POR protein sequences were aligned with MUSCLE [118] and trimmed to remove gaps and ambiguously aligned regions, resulting in a 274 amino acid alignment of 275 sequences representing 162 taxa. Available protein matrices were evaluated for appropriateness using ProtTest 2.4 [119]. The WAG + I + Γ model of protein sequence evolution was found to best suit the data. Trees were inferred in the CIPRES Science Gateway [120] using RAxML 8.0.24 [121] with 1000 bootstraps, as well as MrBayes 3.2.2 [122] with two runs each of four chains, 10,000,000 generations and 25% burn-in. The Whelan and Goldman matrix for globular proteins (WAG, [55]), a Gamma shape parameter, and an empirical estimation of invariable sites was used for both the Bayesian and maximum-likelihood analyses. Stationarity and convergence of the Bayesian analysis were assessed with Tracer v1.5 [123]. Maximum likelihood and Bayesian methods recovered nearly identical topologies (see Figures 3B, 4). Trees were visualized in FigTree [124].

### Multiple sequence alignment (MSA) sequence logo construction

Cyanobacterial and diatom POR protein sequences used in the POR gene tree were aligned with MUSCLE [118] and incomplete sequences were removed, resulting in an alignment of 18 cyanobacterial sequences (18 genera) and 21–22 diatom sequences (15–16 genera) (Additional file 4). The alignment was trimmed to the N-terminus of the cyanobacterial POR proteins and gaps pertaining to two or fewer sequences were removed. Numbering is in accordance with the cyanobacterium *Synechocystis elongatus* (YP_401520) and the diatom *Phaeodactylum tricornutum* (XP_002179689; XP_002180992).

## Availability of supporting data

Gene sequences obtained in course of this study have been deposited in GenBank under accessions KJ408437-45. The data sets supporting the results of this article are available in the Dryad repository at http://dx.doi.org/10.5061/dryad.3ss6p [125].

## Additional files

Additional file 1:
**POR protein alignment for phylogenetic inference.** FASTA format (.fa). Additional file 2:
**Bayesian phylogenetic tree.** Nexus file (.nex), formatted for FigTree [124]. Additional file 3:
**Maximum-likelihood tree.** Newick format (.tre). Additional file 4:
**Complete list of POR protein sequences.** Excel spreadsheet (.xlsx) of identifying information of POR protein sequences used, organized by taxon and clade in the POR phylogeny. Additional file 5:
**Sequencing primers.** Excel spreadsheet (.xlsx) listing degenerate and species-specific primers used to amplify and sequence *por* genes.

## Abbreviations

CASH: Assemblage comprising Cryptophyte, Alveolate (dinoflagellate), Stramenopile and Haptophyte algae
EGT: Endosymbiotic gene transfer
HGT: Horizontal gene transfer
LIPOR: Light-independent protochlorophyllide oxidoreductase
Pchlide: Protochlorophyllide
POR: Light-dependent protochlorophyllide oxidoreductase
UTC: Ulvophyte-trebouxiophyte-chlorophyte
